# Use of Gabapentin in the Treatment of Substance Use and Psychiatric Disorders: A Systematic Review

**DOI:** 10.3389/fpsyt.2019.00228

**Published:** 2019-05-07

**Authors:** Saeed Ahmed, Ramya Bachu, Padma Kotapati, Mahwish Adnan, Rizwan Ahmed, Umer Farooq, Hina Saeed, Ali Mahmood Khan, Aarij Zubair, Iqra Qamar, Gulshan Begum

**Affiliations:** ^1^Nassau University Medical Center, East Meadow, NY, United States; ^2^Department of Internal Medicine, Baptist Health-UAMS, Little Rock, AR, United States; ^3^Manhattan Psychiatric Center, New York, NY, United States; ^4^McMaster University, Hamilton, ON, Canada; ^5^Liaquat National Medical College, Karachi, Pakistan; ^6^John T. Mather Memorial Hospital, Port Jefferson, NY USA; ^7^Baqai Medical University, Karachi, Pakistan; ^8^University of Texas Rio Grande Valley Edinburg, Edinburg, TX, United States; ^9^St. John’s University, Queens, NY, United States; ^10^Department of Cardiology Brigham & Women’s Hospital, Boston, MA, United States; ^11^Department of Psychiatry, Interfaith Medical Center, Brooklyn, NY, United States

**Keywords:** gabapentin, neurontin, bipolar disorder, substance use disorder, alcohol use disorder, alcohol withdrawal, PTSD, anxiety disorder

## Abstract

**Objective:** Gabapentin (GBP) is an anticonvulsant medication that is also used to treat restless legs syndrome (RLS) and posttherapeutic neuralgia. GBP is commonly prescribed off-label for psychiatric disorders despite the lack of strong evidence. However, there is growing evidence that GBP may be effective and clinically beneficial in both psychiatric disorders and substance use disorders. This review aimed to perform a systematic analysis of peer-reviewed published literature on the efficacy of GBP in the treatment of psychiatric disorders and substance use disorders.

**Methods:** This review was performed according to the Preferred Reporting Items for Systematic Reviews and Meta-Analyses (PRISMA) guidelines. The PubMed and Ovid MEDLINE literature databases were screened and filtered by using specific search terms and inclusion/exclusion criteria. The full texts of selected studies were subsequently retrieved and reviewed. The search terms generated 2,604 results from the databases. After excluding all duplicates, 1,088 citations were left. Thereafter, we applied inclusion and exclusion criteria; a total of 54 papers were retained for detailed review.

**Results:** This literature review concludes that GBP appears to be effective in the treatment of various forms of anxiety disorders. It shows some effectiveness in bipolar disorder as an adjunctive therapeutic agent, while the evidence for monotherapy is inconclusive. In substance use disorders, GBP is effective for acute alcohol withdrawal syndrome (AWS) with mild to moderate severity; it reduces cravings, improves the rate of abstinence, and delays return to heavy drinking. GBP may have some therapeutic potential in the treatment of opioid addiction and cannabis dependence, but there is limited evidence to support its use. No significant benefit of GBP has been conclusively observed in the treatment of OCD, PTSD, depression, or cocaine and amphetamine abuse.

**Conclusion:** GBP appears to be effective in some forms of anxiety disorders such as preoperative anxiety, anxiety in breast cancer survivors, and social phobia. GBP has shown to be safe and effective in the treatment of alcohol dependence. However, the literature suggests that GBP is effective as an adjunctive medication rather than a monotherapy. More clinical trials with larger patient populations are needed to support gabapentin’s off-label use in psychiatric disorders and substance use disorders. It is worth noting that numerous clinical studies that are discussed in this review are open-label trials, which are inherently less rigorously analyzed. Therefore, more extensive investigations are required to examine not only the efficacy of GBP, but also its safety and tolerance.

## Introduction

The Food and Drug Administration (FDA) in the United States (US) first licensed gabapentin (GBP) in 1993 as an adjunctive treatment for partial seizures. In 2000, GBP was approved for treatment of partial seizures in children aged 3 years or older. It was subsequently discovered that GBP has analgesic properties and was licensed by the FDA in 2002 for the treatment of post-herpetic neuralgia (1, 2). GBP has been used to treat various medical and psychiatric conditions such as fibromyalgia, chronic pain syndromes, and migraine headaches, and has been extensively prescribed off-label for psychiatric disorders despite a lack of rigorous evidence of its effectiveness (1, 2). GBP belongs to the gabapentinoid class, used as anticonvulsants, analgesics, and anxiolytics among other indications. Although GBP was originally developed to treat epilepsy, it is now also used to relieve neuropathic pain and restless leg syndrome. It is particularly recommended as a first-line agent for the treatment of neuropathic pain arising from diabetic neuropathy, post-herpetic neuralgia, and central neuropathic pain.

However, there is mounting evidence that GBP may also be effective for treating various psychiatric disorders and substance use disorders (1, 2). With growing off-label use, the efficacy of this drug needs to be established *via* clinical studies and other scientific shreds of evidence. The primary objective of this review is to perform a thorough assessment of published studies on the effectiveness of GBP in the treatment of various psychiatric disorders and substance use disorders.

### GBP Pharmacology

GBP is a structural analog of γ-aminobutyric acid (GABA). It demonstrates little or no interaction with GABA receptors and does not appear to alter GABA uptake or metabolism. While initially believed to act on the GABAergic neurotransmitter system, its actual mechanism of action as an anticonvulsant and therapeutically for neuropathy, is unknown. In human and rat studies, GBP was found to increase GABA biosynthesis and to increase non-synaptic GABA neurotransmission *in vitro*. As the GABA system is the most prolific class of inhibitory receptors within the brain, its modulation results in the sedating or calming effects of GBP on the nervous system. GBP has also been shown to bind to the α2δ-1 subunit of voltage-gated calcium ion channels, which contributes to its analgesic effects. It is uncertain how this may contribute to the psychoactive effects of GBP (3).

GBP does not appear to bind to any proteins, nor does it induce or inhibit hepatic microsomal enzymes. Instead, it is eliminated unchanged by renal excretion. GBP offers a favorable safety profile with minimal reported drug–drug interactions. It is not associated with hematologic or hepatic problems and does not require serum concentration monitoring. In addition to these pharmacological advantages, animal experiment data support the use of GBP for the treatment of psychiatric disorders.

## Materials and Methods

The review was performed and reported according to the PRISMA guidelines (4). Two electronic databases, PubMed and Ovid MEDLINE, were scanned from the earliest published articles until those published by the end of November 2018. Studies were selected based on the following inclusion criteria: 1) publication in English, 2) human study population, 3) longitudinal, cohort, case–control, cross-sectional studies that investigated GBP for the treatment of psychiatric disorders and/or substance use disorders, and 4) publication in peer-reviewed journals. Previously published systematic reviews, case series, case reports, opinions, comments, and unpublished studies were excluded from this review.

### Search Terms

In our search we used the following keywords through electronic databases, PubMed and Ovid MEDLINE: 1) Gabapentin, 2) Gabapentin AND Bipolar Disorder, 3) Gabapentin AND Major Depressive Disorder “MDD,” 4) Gabapentin AND Anxiety Disorder, 5) Gabapentin AND Mood Disorder, 6) Gabapentin AND Posttraumatic Stress Disorder “PTSD,” 7) Gabapentin AND Obsessive-Compulsive Disorder “OCD,” 8) Gabapentin AND Alcohol Use Disorder “AUD,” 9) Gabapentin AND “Alcohol abuse” “dependence” “withdrawal” “cravings,” 10) Gabapentin AND “drug abuse” OR “drug dependence,” 11) Gabapentin AND Opioid Use Disorder “OUD” “dependence” “withdrawal” “cravings,” 12) Gabapentin cocaine, “dependence” “withdrawal” “cravings,” 13) Gabapentin AND amphetamine abuse “dependence” “withdrawal” “cravings,” and 14) Gabapentin AND cannabis, OR Marijuana, “dependence” “withdrawal” “cravings.” This search was similarly performed with the other commonly used name “Neurontin”.

The search was carried out by two independent volunteer researchers (PK and MA). These researchers independently performed screening of titles and abstracts for initial exclusion based on publication type. Where required, the full texts were immediately screened to ascertain inclusion or exclusion. Following the initial exclusion screening, the researchers further evaluated the full-text articles for inclusion based on the four inclusion criteria. At each step, their results were compared, and any discrepancies were resolved through discussion. Any remaining disagreements were resolved by involving a third independent researcher (SA).

## Results

**Figure 1:**
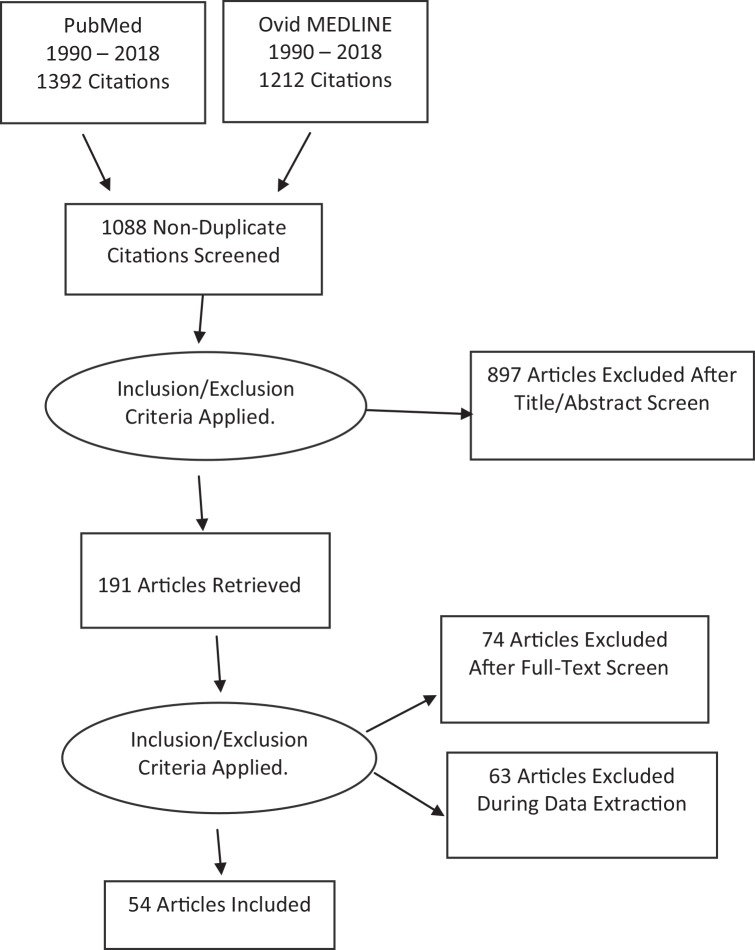
Literature mining and screening process of articles related to gabapentin in the treatment of substance use and psychiatric disorders.

Our search of the PubMed and Ovid MEDLINE databases generated 2,604 results. After excluding all duplicates and completely off-topic titles, 1,088 citations were left. Remaining citations were manually screened for exclusion based on their titles and abstracts, i.e., those clearly incompatible with the purpose of our review, and designed four eligibility criteria such as articles that were written in languages other than English, gray literature/unpublished/non-peer-reviewed papers. A manual search of references revealed 190 additional articles. Subsequently, full-text articles were obtained for those 191 references that appeared to match our inclusion criteria. Out of those, 137 papers did not fit the inclusion criteria. A final total of 54 articles were found for the purpose of the study (**Figure 1**).

### Anxiety Disorders

The annual prevalence of anxiety disorders among the adult (∼18 years and older) population in the US is about 40 million (5). Despite the high prevalence, anxiety disorders are frequently underdiagnosed and undertreated by primary care physicians. Untreated patients of anxiety disorders may also suffer from depression, distressing physical signs, substance abuse, and socioeconomic problems. These subsequently cause decreased functionality, increased morbidity, and mortality rates.

Anxiety disorders can be effectively treated with psychopharmacological and psychotherapeutic interventions such as cognitive–behavioral interventions. Drugs prescribed for the treatment of anxiety disorders include selective serotonin reuptake inhibitors (SSRIs), serotonin and norepinephrine reuptake inhibitors (SNRIs), benzodiazepines not used for first-line treatment because of side effects, primarily used for acute anxiety, and tricyclic antidepressants (TCAs). Other drugs, such as hydroxyzine, mirtazapine, nefazodone, and atypical neuroleptic agents, have also been used (6). A number of antiepileptic drugs (AEDs) have been used for anxiety disorder. The results for the use of GBP in anxiety disorder are somewhat mixed; some studies have found it to be useful in patients with social phobias, preoperative anxiety, and moderate to severe panic disorder.

To the best of our knowledge, there have been 11 clinical trials (RCTs), comprising a total of 1,230 patients, to determine the efficacy of GBP in the treatment of various forms of anxiety disorders (7–17) (**Table 1**). In a randomized, double-blinded controlled trial, Lavigne et al. (10) compared 300 mg GBP versus 900 mg GBP versus placebo in 420 breast cancer patients. At 4 weeks, anxiety change scores were significantly better for GBP 300 and 900 mg (*p* = 0.005) compared to placebo. At 8 weeks, the anxiolytic effects of GBP compared to placebo persisted (*p* < 0.005). Researchers found GBP to be an effective agent for hot flashes and anxiety (10). Pande et al. (12) studied the efficacy and safety of GBP in relieving the symptoms of social phobia. Sixty-nine patients were randomly assigned to receive double-blind treatment with either GBP (dosed flexibly between 900 and 3,600 mg daily in three divided doses) or placebo for 14 weeks. A significant reduction (*p* < 0.05) in the symptoms of social phobia was observed among patients taking GBP compared with those on placebo (12). The same researchers conducted a study in the following year to see the efficacy and safety of GBP in patients with panic disorder. Pande et al. (13) studied 103 patients, who were randomly assigned to GBP (dosed flexibly between 600 and 3,600 mg/day) or placebo for 8 weeks. Results from this study show that GBP appeared to be less effective for persistent and severe anxiety because of no significant overall difference between the placebo and the GBP group (13). However, in subsequent years, another study demonstrated the efficacy of GBP in treating patients with panic disorder. Quevedo et al. (14) examined the anxiolytic potential of GBP in those who suffer from anxiety induced by simulated public speaking (SPS). Thirty-two normal male volunteers (aged 17–30 years) had their anxiety and mood evaluated by self-scales [Visual Analogue Mood Scale (VAMS) and Profile of Mood State (POMS)] during the SPS procedure. Treatment with GBP at 800 mg significantly decreased the anxiety of subjects by showing a decrease in the VAMS item calm–excite and also volunteers who received GBP 400 and 800 mg showed a decrease in hostility score in POMS. The study also suggested that GBP may offer a good alternative to panic disorder treatment with fewer side effects and overall better safety profile (14).

**Table 1:** Gabapentin and psychiatric disorders.

Author/year/type of study	Diagnosis	*N*	Duration	GBP dose	Placebo/adjunctive medication	Outcome measures	Results
Pande et al./1999/RCT (12)	Anxiety disorder	69	14 weeks	900–3,600 mg/day in three divided doses	Placebo	Clinician- and patient-rated scales	GBP significantly reduced (*p* < 0.05) the symptoms of social phobia compared with the placebo.
Pande et al./2000/RCT (13)	Anxiety disorder	103	8 weeks	600–3,600 mg/day	Placebo	PAS	No significant difference (*p* = 0.606) in PAS scores between GBP and placebo groups in the treatment of panic disorder.
Lavigne et al./2012/RCT (10)	Anxiety disorder	420	8 weeks	300 mg/day, 900 mg/day	Placebo	STAI	STAI scores for breast cancer survivors at 4 weeks were significantly better for the GBP-treated group (both 300 and 900 mg/day; *p* = 0.005) compared to the placebo. Anxiolytic effects of GBP persisted at 8 weeks compared to the placebo (*p* < 0.005). No significant interactions observed.
Ménigaux et al./​2005/RCT (11)	Anxiety disorder	40	GBP given before knee surgery	1,200 mg	Placebo	VAS	GBP significantly reduced preoperative anxiety (*p* < 0.001) and provided significant postoperative analgesia (*p* < 0.001) after knee surgery.
Khezri et al./2013/RCT (9)	Anxiety disorder	130	GBP given before cataract surgery	600 mg	Placebo/melatonin (6 mg)	VAS	Anxiety scores showed a significant difference in the GBP-treated group compared to the placebo group after premedication (*p* = 0.005). No statistical difference in anxiety between melatonin and GBP groups. A significant difference (*p* = 0.046) in sedation scores during retrobulbar block placement between GBP and placebo groups (no difference between melatonin and GBP).
Tirault et al./2010/RCT (15)	Anxiety disorder	210	GBP given preoperatively	1,200 mg	Placebo/hydroxyzine (75 mg)	100-mm VAS	Anxiety in the GBP group was significantly lower in the holding area and before induction of anesthesia than in the hydroxyzine and placebo groups. Anxiety decreased significantly over time only in the GBP group (*p* = 0.006).
Adam et al./2012/RCT (7)	Anxiety disorder	64	GBP given preoperatively	1,200 mg	Placebo	STAI, VAS	STAI decreased significantly in the GBP group and remained unchanged in the placebo group (*p* = 0.003). The VAS score for anxiety also decreased, but not significantly, in the GBP and control groups.
Clarke et al./2010/RCT (8)	Anxiety disorder	70	GBP given preoperatively	600 mg	Placebo	VAS	Anxiety scores did not differ significantly between the GBP and placebo groups either before or 2 h after GBP/placebo ingestion.
Clarke et al./2012/RCT (17)	Anxiety disorder	44	GBP given preoperatively	1,200 mg	Placebo	Numeric rating scale (NRS) anxiety score	Analysis of covariance in which pre-drug NRS anxiety scores were used as the covariate showed that post-drug preoperative NRS anxiety [effect size, 1.44; confidence interval (CI), 0.19 to 2.70] and pain catastrophizing (effect size, 0.43; CI 0.12 to 0.74) scores were significantly lower in the gabapentin group than in the placebo control group, respectively.
Pathak and Chaturvedi/2012/RCT (16)	Anxiety disorder	80	GBP given preoperatively	1,200 mg	Placebo	VAS	GBP premedication prior to cholecystectomy significantly reduced preoperative anxiety and postoperative pain compared to the placebo.
Quevedo et al./2003/open label (14)	Anxiety disorder	32	GBP given after simulated public speaking	400 mg, 800 mg	No control	VAMS, POMS	In subjects exposed to public speaking, VAMS score decreased for those treated with 800 mg of GBP. POMS hostility score decreased when subjects treated with 400 and 800 mg of GBP.
Önder et al./2008/randomized open label trial (20)	Obsessive–compulsive disorder	40	8 weeks	600–900 mg/day	Fluoxetine (40–60 mg/day)	Y-BOCS, CGI	CGI and Y-BOCS scores were not significantly different between the GBP plus fluoxetine group and the fluoxetine-only group at weeks 4, 6, and 8 of treatment.
Pande et al./2000/RCT (36)	Bipolar disorder	114	10 weeks	900–3,600 mg/day	Placebo	YMRS, HDRS	YMRS scores decreased in both treatment groups from baseline to endpoint, but this decrease was significantly greater for the placebo group (*p* < 0.05).
Obrocea et al./2002/RCT (50)	Bipolar disorder	45	6 weeks	4,800 mg/day	Placebo/lamotrigine (500 mg/day)	CGI-BP	Response to lamotrigine (51%) exceeded that to GBP (28%) and the placebo (21%) GBP.
Frye et al./2000/RCT (36)	Bipolar disorder	31 (refractory bipolar and unipolar mood disorders)	6 weeks	4,800 mg/day	Placebo/lamotrigine (500 mg/day)	CGI	CGI scores were significantly improved (*p* = 0.031) for lamotrigine only: LTG, 52% (16/31); GBP, 26% (8/31); and placebo, 23% (7/31). No difference observed between the GBP and placebo groups.
Vieta et al./2006/RCT (27)	Bipolar disorder	25 (in remission with bipolar I and II disorder)	1 year	900–2,400 mg/day	Placebo	CGI-BP, YMRS, HDRS, HARS, PSQI	CGI-BP scores were reduced to a greater extent (*p* = 0.0046) in the GBP group versus the placebo group from baseline to endpoint.
Perugi et al./1999/open label (30)	Bipolar disorder	21	8 weeks	300–2,000 mg/day	Mood stabilizers	HRDS, YMRS, CGI	GBP may be useful in the adjunctive treatment of drug-resistant bipolar mixed states and may be particularly effective in relation to depressive symptomatology.
Astaneh and Rezaei/​2012/open label (23)	Bipolar disorder	60	6 weeks	900 mg/day	Lithium	YMRS	YMRS was significantly improved in the GBP and lithium group compared to the lithium only group (*p* < 0.001)
Erfurth et al./1998/open label (33)	Bipolar disorder	14	21 days	1,200–4,800 mg/day	None	BRMAS	GBP monotherapy may be useful in selected patients to treat modest and non-severe manic states. Also, GBP in conjunction with other effective mood stabilizers appears to be safe and effective in the treatment of severe mania.
Vieta et al./2000/open label (31)	Bipolar disorder	22	12 weeks	GBP dose increased until a clinical response	Mood stabilizers	CGI-BP	The results suggested that GBP is a useful drug for adjunctive treatment of bipolar patients with poor response to mood stabilizers.
Young et al./1999/open label (28)	Bipolar disorder	37	6 months	600–2,400 mg/day	Mood stabilizers	HAM-D, YMRS, CGIS	GBP significantly reduced acute mania and depressive symptoms.
Wang et al./2002/open label (32)	Bipolar disorder	22	12 weeks	1,725 mg/day	Mood stabilizers	HDRS, YMRS, CGI-S	Overall HDRS significantly decreased from 32.5 ± 7.7 to 16.5 ± 12.8 (*p* < 0.0001). Adjunctive GBP treatment was effective in treating mild to moderate depression.
Altshuler et al./1999/open label (35)	Bipolar disorder	28	GBP given until clinical response	600–3,600 mg/day to patients not responsive to mood stabilizers	Mood stabilizers	CGI-BP	Patients with hypomania responded fastest (12.7 ± 7.2 days). Patients with classic mania had a mean time to positive response of 25 ± 12 days, and patients with mixed mania took 31.8 ± 20.9 days to respond. Patients with depression had a positive response within 21± 13.9 days.
Cabras et al./1999/open label (27)	Bipolar disorder	22	16 weeks	1,440 mg/day	Mood stabilizers, antipsychotics	CGI, BPRS	Adjunctive GBP was effective in treating mania and hypomania in patients with bipolar disorder or schizoaffective disorder.
Sokolski et al./1999/open label (35)	Bipolar disorder	10	30 days	300–600 mg/day	Mood stabilizers	HAM-D, BMR	Reduced Hamilton Depression (*p* < 0.05) and Beck Mania Ratings (*p* < 0.01) were evident in the first week of treatment and were sustained. GBP may, therefore, be beneficial for bipolar patients who only respond partially to other mood stabilizers.
Stein et al./2007/RCT (45)	Posttraumatic stress disorder	48	14 days	900–1,200 mg/day	Placebo/propranolol	ASDS, CIDI	Neither study drug showed a significant benefit compared to the placebo for the treatment of depressive or posttraumatic stress symptoms

Out of 10 clinical trials from our literature search, 7 have assessed preoperative anxiety in individuals otherwise not affected by severe anxiety (7–9, 11, 14, 16, 17). Six of these seven trials showed that GBP significantly reduced anxiety compared to the placebo, and in some studies, GBP was found to reduce postoperative pain (7, 9, 11, 14, 16, 17). For example, in Ménigaux et al.’s RCT, 40 patients were given 1,200 mg of GBP preoperatively, and the preoperative anxiety scores were significantly lower (*p* < 0.001) in the GBP group compared to the control group (11). The GBP-treated group also required less morphine than the control group (*p* < 0.001), and the patients’ visual analog scale (VAS) pain scores at rest and after mobilization were significantly reduced (*p* < 0.001) in the GBP group. In addition, the first and maximal passive and active knee flexion, at 24 and 48 h, was substantially more extensive in this group. Similarly, a study by Clerk et al. (2012) shows that administration of GBP prior to surgery reduces preoperative anxiety scores and pain catastrophizing scores. These results suggest that GBP may be a useful treatment option for those patients who exhibit high levels of preoperative anxiety and pain catastrophizing; however, the sedative properties of GBP can delay in postoperative discharge (17). The other studies have similarly shown a positive effect of GBP on preoperative anxiety and pain, e.g., Khezri et al. (9), Tirault et al. (15), Adam et al. (7), and Pathak and Chaturvedi (16). Contrary to these results, Clarke et al. concluded in their study that there are no significant differences in the preoperative anxiety levels (8). In summary, GBP has been shown to be an effective preoperative anxiety treatment despite not being a first-line medication.

### Obsessive–Compulsive Disorder

Obsessive–compulsive disorder (OCD) is a common psychiatric illness with a lifetime prevalence of 1−3%. It is the fourth most common psychiatric illness and a leading cause of disability. OCD can cause significant impairment in functioning and quality of life. If untreated, OCD can develop into a chronic illness with waxing and waning symptoms (18). The mainstay of treatment for OCD includes medications and cognitive behavioral therapy (CBT) in the form of exposure and response prevention (ERP). SSRIs are typically used as first-line therapeutics with SNRIs and TCAs as second-line therapeutics. Where SSRI treatment alone is ineffective, augmentation of SSRIs with antipsychotics is recommended (19). Our literature search identified only one randomized study that has been carried out to date to assess the effect of GBP on OCD. Önder et al. randomized a group of 40 subjects; fluoxetine was given to half of the subjects, while fluoxetine and GBP were given to the other half (20). The results were assessed with the Clinical Global Impression (CGI) and Yale–Brown Obsessive–Compulsive Scale (Y-BOCS), which showed no significant difference between these two groups over time. This study shows that GBP shortens flouxetine’s onset of action without a significant increase in adverse effects. Authors suggested that GBP can be used as adjunctive to accelerate the clinical response of fluoxetine. Other than this one study, there are few clinical trials examining the effects of GBP in OCD, thereby impeding conclusions about the efficacy of its use in this group of disorders.

### Bipolar Disorder

Bipolar disorder affects more than about 5.7 million US adults, which accounts for about 2.6% of the population that is 18 years and older (21, 22). The lifetime prevalence of bipolar disorder is approximately 1% with equal prevalence in both men and women and a documented 40–50% of patients experiencing their second episode of mania within 2 years of their first episode (23). Treatment of bipolar disorder is broadly classified as mood stabilizers, antipsychotic medications, anticonvulsants, antidepressants, electroconvulsive therapy (ECT), and psychosocial interventions. Use of appropriate medication for managing bipolar disorder, or manic-depressive illness (MDI), depends on the phase of illness in which a patient presents to the clinician and their past treatment history. Treatment of bipolar disorder generally has two phases. 1) Acute phase treatment is mainly focused on the management of acute mood episodes such as manic, hypomanic, or depressive. 2) The second phase is the maintenance phase treatment, which is focused on preventing recurrences of acute episodes.

The mainstay of management of bipolar disorder is mood stabilizers. Mood stabilizers (lithium and divalproex) are the first-line treatments for acute mania and mixed episodes, with second-generation antipsychotics (aripiprazole, asenapine, olanzapine, quetiapine, risperidone, and ziprasidone) used for subsequent treatment. Acute bipolar depression is treated with first-line medications such as quetiapine, lamotrigine, or a combination of olanzapine and fluoxetine, and the second-line treatments comprise lithium, divalproex, and combinations of SSRIs or bupropion with the second-generation antipsychotics. Maintenance therapy includes lithium and divalproex, carbamazepine, lamotrigine, and second-generation antipsychotics. Psychoeducation focusing on the recognition of early warning signs of relapse is an effective adjunct to medication and should be offered to all patients with bipolar disorder (24).

Psychotherapies also play a crucial role in the treatment of bipolar disorders by optimizing disease stability and psychosocial functioning. Psychotherapies target essential therapeutic areas such as lack of support, acceptance and improving understanding of illness and its management, medication adherence, managing interpersonal issues, and lastly, identifying and responding to early signs of mood episode relapse (25, 26).

GBP has been used as both monotherapy and adjunctive therapy in bipolar disorders. Several RCTs and open-label trials have assessed the efficacy of GBP in patients with bipolar disorder (**Table 1**). The majority of these studies, e.g., Vieta et al. (27), Young et al. (28), and Cabras et al. (29), showed that GBP was effective in treating bipolar disorder. However, GBP appears to be effective as an adjunctive medication rather than as a monotherapy (23, 29–32). In the late 1990s, initial studies on the efficacy of GBP in bipolar disorder showed that GBP seems to exert moderate antimanic properties. Among those studies, Erfurth et al. suggested that the effectiveness of GBP for monotherapy is higher in modest and nonsevere manic states, while GBP in conjunction with other effective mood stabilizers appears to be safe and effective for the treatment of severe mania (33). Sokolski et al. (34), Altshuler et al. (35), and Wang et al. (32) also demonstrated that GBP might be particularly useful in the treatment of drug-resistant bipolar disorder in relation to residual depressive symptoms such as irritability, social withdrawal, and anxiety. Contrary to these studies, two studies from the same era, Pande et al. (36) and Frye et al. (37), concluded that GBP has no superiority to the placebo or lamotrigine in bipolar patients. The studies until a decade ago showed overall success in the management of bipolar disorder, although our literature search couldn’t find studies, especially at larger scale over the past decade, documenting similar success.

### Posttraumatic Stress Disorder

Posttraumatic stress disorder (PTSD) is a common psychiatric disorder with a 12‐month and lifetime prevalence of 10.1% and 3.7%, respectively, in the US general population. The lifetime prevalence of PTSD among women (10.4%) makes them twice as likely as men (5%) to have the disorder at some point in their lives (38).

Studies recommend various psychotherapy interventions, particularly trauma-focused psychotherapy, as the first-line treatment for PTSD over pharmacotherapy. The American Psychological Association’s guidelines suggest evidenced-based psychotherapies such as CBT, cognitive processing therapy (CPT), cognitive therapy (CT), prolonged exposure therapy (PE), brief eclectic psychotherapy (BEP), eye movement desensitization and reprocessing (EMDR), and narrative exposure therapy (NET) (39). The commonly used medications with strong evidence for the treatment of PTSD are SSRIs) (e.g., sertraline, paroxetine, and fluoxetine) and selective SNRIs) (e.g., venlafaxine) (39). Currently, only sertraline and paroxetine are approved by the FDA for PTSD (39–41). While SSRIs are typically the first class of medications used in PTSD treatment, exceptions exist for patients based on their individual histories of side effects, drug response, comorbidities, and personal preferences. Several AEDs such as carbamazepine, lamotrigine, and valproate have been used in the treatment of PTSD and have shown effectiveness for the treatment of PTSD (42). Given GBP’s potential efficacy in anxiety disorders, alcohol withdrawal, bipolar disorder, and behavioral disorders, it was studied in PTSD patients as well. Published literature suggests that GBP may exert its effects through its structural relationship to GABA, playing an essential role in decreasing excitatory input of glutamate at the *N*-methyl-d-aspartate (NMDA) receptors as well as α-amino-3-hydroxy-5-methyl-4-isoxazolepropionic acid (AMPA) receptors. In turn, it plays a role in sensory transmission important in the psychobiology of PTSD (43, 44). Only one randomized study has been published to date on its use for this indication. Stein et al. assessed the effect of GBP on PTSD in 48 subjects; 17 patients received propranolol (60–120 mg/day), 14 patients received GBP (900–1,200 mg/day), and 17 patients received the placebo within 48 hours of trauma (45). The results were assessed at 8 months post-trauma. Neither drug conferred any significant reduction in PTSD symptoms. However, a review of 30 PTSD patients showed GBP was useful to facilitate sleep, as the majority (77%) of patients showed moderate or greater improvement in duration of sleep and most noted a decrease in the frequency of nightmares (46).

### Depression

Major depression is a leading cause of disability in the US and worldwide. Great disparities exist in access and utilization of mental health services for depressive disorders in the Hispanic and African American population, especially when compared to their Caucasian counterparts (47). Based on the 2012 US census, a population of more than 16 million live with major depressive disorder (MDD) in the US, with a global estimate of 350 million people with MDD (47). Generally, medications and psychotherapy are effective for most people with depression. Current recommended medications include SSRIs, SNRIs, TCAs, and atypical monoamine oxidase inhibitors (MAOIs). ECT is limited to patients who are either highly resistant to treatment or psychotic and for whom the efficacy of other somatic therapies has not yet been established. MDD treatment requires patience on the patient’s behalf, as medications can require several weeks or longer to take full effect and for the side effects to ease as the body adjusts. Different forms of psychotherapy can also be effective in the treatment of depression; these include CBT and interpersonal therapy (48).

To the best of our knowledge, there are no randomized trials assessing the efficacy of GBP in the treatment of MDD. However, patients with epilepsy with concurrent depressive symptoms have been studied. A prospective nonrandomized study selected 40 adults with partial epilepsy for treatment with GBP (*N* = 20; 615 mg/day) or the control (*N* = 20) for 3 months, and the outcomes were measured with the Cornell Dysthymia Rating Scale (CDRS), Beck’s Depression Inventory (BDI), as well as the Hamilton Depression (HAM-D) and Anxiety (HAM-A) scoring. GBP significantly decreased the CDRS score over time compared to the control (*p* = 0.04), and GBP treatment was associated with mood improvement as measured by the CDRS (49). No significant differences were found with any of the other scales. Again, there is a lack of large-scale, robust trials to study the effects of GBP on depressive disorders; thus, not much is known about its application for this psychiatric disorder.

### Substance Use Disorders

#### i Alcohol Withdrawal and Dependence

Alcohol use disorders, which include both alcohol abuse and dependence, make up one of the most prevalent categories of substance use disorders (51). Three medications are approved by the FDA to treat alcohol use disorder: naltrexone, acamprosate, and disulfiram. A fourth drug named “nalmefene” is approved throughout the European Union and is used in an “as needed/pro renata/PRN” basis prior to anticipated drinking occasions. Several randomized studies have assessed the role of GBP in the treatment of alcohol abuse or dependence. Our literature search found 13 trials comprising a total of 807 patients (52–64) (**Table 2**). Published studies showing reduced drinking, fewer withdrawals, minimized cravings, and decreased alcohol-related insomnia illustrate the therapeutic potential of GBP in alcohol use disorders. The majority of studies have shown that relatively high doses of GBP 1,200–3,200 mg/day may have a positive effect on alcohol withdrawal symptoms, cravings, sleeplessness, depression, and in maintaining abstinence (57, 58). Insomnia is also commonly associated with relapse in alcohol-dependent patients, with rates ranging from 36% to 91%. One can hypothesize that treating insomnia in these patients may decrease relapse rates. In such cases, GBP has been shown to address insomnia and delay relapses in alcohol-dependent patients (57).

**Table 2:** Gabapentin and substance use disorders.

Author/year/type of study	Diagnosis	*N*	Duration	GBP dose	Placebo/adjunctive medication	Outcome measures	Results
Bonnet et al./2003/RCT (55)	Alcohol withdrawal	61	7 days	1,600 mg/day	Placebo/clomethiazole	CIWA	GBP was not superior to the placebo in reducing the dose of CLO required to treat acute AWS (*p* = 0.96).
Anton et al./2009/RCT (52)	Alcohol withdrawal	60	8 weeks	1,200 mg/day	Placebo/flumazenil	PDA, TFHD	Flumazenil and GBP adjunctively treated group were superior to the placebo in patients with more AW symptoms.
Anton et al./2011/RCT (53)	Alcohol withdrawal	150	16 weeks	1,200 mg/day	Placebo/naltrexone	SCID-IV, ADS, OCDS, POMS	GBP plus naltrexone was superior to naltrexone only and the placebo in reducing drinks/day (*p* = 0.01) and heavy drinking days (*p* = 0.0002) during the first 6 weeks. The effect did not persist over the remaining weeks once GBP was discontinued.
Myrick et al./2009/RCT (57, 62)	Alcohol withdrawal	100	12 days	900–1,200 mg/day	Lorazepam	CIWA-AR, Breath alcohol levels	GBP was superior to lorazepam at higher doses (*p* = 0.09).
Stock et al./2013/RCT (58, 63)	Alcohol withdrawal	26	6 days	300–1,200 mg/day	None/chlordiazepoxide	ESS, PACS, ataxia rating, CIWA-AR	There was no difference between the GBP and chlordiazepoxide groups in reducing the CIWA score, but GBP may improve alcohol craving and sedation at the end of detoxification (*p* = 0.04).
Trevisan et al./2008/RCT (59, 64)	Alcohol withdrawal	57	4 weeks	1,200 mg/day	Placebo/valproic acid, lorazepam	CIWA-AR, PSQI, OCDS	Valproic acid and GBP were not significantly superior to the placebo in reducing symptoms of withdrawal and the associated psychiatric symptoms or in preventing relapses.
Bonnet et al./2009/open label (55, 56)	Alcohol withdrawal	37	2 days	3,200 mg/day	Clonazepam	CIWA-AR	GBP loading dose was helpful only for mild AWS and was not beneficial in treating severe AWS.
Myrick et al./2007/RCT (61)	Alcohol dependence	35	8 days	1,200 mg/day	Placebo	CIWA-AR, SCID, ADS, OCDS, SAAST, POMS	GBP was not superior to the placebo in reducing drinking and craving (*p* = 0.19).
Furieri and Nakamura-Palacios/2007/RCT (58)	Alcohol dependence	60	4 weeks	600 mg/day	Placebo	CIWA-AR, MMSE, OCDS	GBP was superior to the placebo in reducing alcohol consumption and craving (*p* = 0.02), which may have helped patients to maintain abstinence (*p* = 0.08).
Mason et al./2009/RCT (59)	Alcohol dependence	33	1 week	1,200 mg/day	Placebo	ACQ, PSQI, TFBI, subjective sleep quality, subjective alcohol craving	GBP was significantly more effective than the placebo in reducing craving (*p* = 0.09) and in improving sleep quality, making it effective for treating the protracted abstinence phase of alcohol withdrawal.
Mason et al./2014/RCT (60)	Alcohol dependence	150	12 weeks	1,800 mg/day	Placebo	TFBI, weekly breath analyzer, GGT, Craving Questionnaire short form, BDIII, PSQI	GBP significantly improved the rate of abstinence and prevented heavy drinking (*p* < 0.01).
Bisaga and Evans/2006/RCT (54)	Alcohol dependence	17	30 days	0, 1,000, and 2,000 mg/day were given in three successive phases	None	ACS, VAS, CADSS	High doses of GBP were tolerated in combination with alcohol and did not alter the effects of alcohol (*p* < 0.01).
Brower et al./2008/RCT (57)	Alcohol dependence	21	12 weeks	1,500 mg/day	Placebo	TLFB method, polysomnography, subjective scale	GBP treatment may result in a delayed return to heavy drinking that persists after 6 weeks of treatment.
Kheirabadi et al./2008/RCT (67)	Opioid dependence	40	3 weeks	900 mg	Placebo	SOWS	No significant differences were reported between the GBP and placebo groups.
Salehi et al./2011/RCT (66)	Opioid dependence	27	3 weeks	1,600 mg/day	Placebo	SOWS	Significant reduction in SOWS scores in the GBP group (*p* = 0.06).
Sanders et al./2013/RCT (70)	Opioid dependence	24	5 weeks	Not given	Placebo	Opioid withdrawal scale, urine drug screens, vitals	GBP significantly reduced (*p* = 0.004) opioid use during a 10-day buprenorphine detoxification procedure.
Moghadam and Alavinia/2013/RCT (69)	Opioid dependence	60	3 weeks	300 mg/day	Placebo	Methadone consumption, withdrawal symptoms	Decreased methadone consumption in the GBP group compared to placebo (*p* = 0.001).
Ziaaddini et al./2015/RCT (71)	Opioid dependence	59	10 days	900 mg/day	Methadone, tramadol	ARSW, COWS, VAS	There was no significant difference in ARSW score (*p* = 0.263) and COWS score (*p* = 0.862) between the GBP plus tramadol group versus the methadone group. Difference in VAS score for craving between the two groups was almost significant (*p* = 0.057).
Kheirabadi et al./2018/RCT (68)	Opiate withdrawal	50	4 weeks	1,600 mg/day	Placebo/pregabalin	SCOWS	Dosages of 450 mg/day of pregabalin and 1,600 mg/day of GBP were not significantly superior to the placebo in reducing opiate withdrawal symptoms.
Berger et al./2005/RCT (73)	Cocaine dependence	60	10 weeks	1,800 mg/day	Placebo/reserpine, lamotrigine	Urine toxicology, CGI, ASI, SUR, BSCS, self-reports of cocaine use	Urine results indicated significant improvement for the reserpine group (*p* < 0.05) and non-significant changes for the other study groups. Subjective measures of cocaine dependence indicated that GBP did not provide any significant improvement.
Bisaga et al./2006/RCT (74)	Cocaine dependence	129	16 weeks	3,200 mg/day	Placebo	Urine toxicology tests; self-reported craving, drug use, and mood	GBP was not superior to the placebo in treating cocaine dependence over the course of 12 weeks of treatment.
González et al./2007/RCT (75)	Cocaine dependence	76	10 weeks	2,400 mg/day	Placebo/tiagabine	Urine screens, self-reported drug use, Addiction Severity Index, SCID, Center for Epidemiologic Studies Depression Inventory	GBP was no different to the placebo (*p* = 0.4) and was inferior to tiagabine (*p* = 0.05) in treating cocaine dependence and methadone-stabilized, treatment-seeking patients.
Mancino et al./2014/RCT (77)	Cocaine dependence	99	12 weeks	1,200 mg/day	Placebo/sertraline	Urine screens, Hamilton depression ratings	There was no significant difference between the sertraline only group and the sertraline plus GBP group (*p* = 0.10).
Raby and Coomaraswamy/2004/open-label (72)	Cocaine dependence	9	20 weeks	800–2,400 mg/day	None	Urine drug screen	GBP appeared to be safe and efficacious in reducing cocaine usage (*p* < 0.01).
Haney et al./2005/open-label (76)	Cocaine dependence	8	47 days	0–1,200 mg/day	None	Cocaine discriminative stimulus effects, performance tasks, cardiovascular measures	The highest dose of GBP (1,200 mg) decreased the discriminative stimulus effect of cocaine and reduced craving (*p* = 0.01); however, there were no changes in psychomotor task performance or the subjective effects of craving. GBP benefit, therefore, was not clinically significant at the dose tested.
Heinzerling et al./2006/RCT (78)	Amphetamine dependence	88	16 weeks	800 mg/day	Placebo/baclofen	Urine sample screening, psychological counseling	GBP was not found to be effective in the treatment of methamphetamine dependence. Baclofen was found to have a small beneficial effect relative to the placebo.
Urschel III et al./2011/RCT (80)	Amphetamine dependence	135	30 days	1,200 mg/day	Flumazenil	Urine sample screening, self-reported drug use, craving scales	Craving was significantly reduced in the flumazenil plus GBP group compared to the placebo group following the initial treatment period and throughout the 30 days (*p* < 0.01). Decreased methyl amphetamine (*p* < 0.01) use was also observed, as measured by urine drug screens and self-reports.
Ling et al./2011/RCT (79)	Amphetamine dependence	120	108 days	1,200 mg day	Flumazenil, hydroxyzine	Urine sample screening, BSCS, ASI, SCID, IV-TR	PROMETA protocol was not found to be superior to the placebo in reducing craving, improving treatment retention, or reducing drug use (*p* = 0.71).
Mason et al./2012/RCT (85)	Cannabis dependence	50	12 weeks	1,200 mg/day	Placebo	Urine drug screening, timeline follow-back interviews, marijuana withdrawal checklist	Relative to placebo, GBP significantly reduced cannabis use as measured both by urine toxicology (*p* < 0.001) and by the Timeline Follow-back Interview (*p* = 0.004) and significantly decreased withdrawal symptoms as measured by the Marijuana Withdrawal Checklist (*p* < 0.001). GBP was also associated with significantly greater improvement in overall performance on tests of executive function (*p* = 0.029).

An interesting set of studies are the two trials by Mason et al. in 2009 and 2014, which examined the effect of GBP in alcohol dependence. These studies sought evidence for the effectiveness of GBP in supporting recovery from alcohol dependence, through the treatment of symptoms related to protracted abstinence that may trigger relapses (59). The first study in the year 2009 by Mason et al. concluded that GBP might be an effective treatment modality for the protracted abstinence phase in alcohol dependence. In the second study in the year 2014, Mason et al. investigated the safety and efficacy of GBP with a higher dose of GBP (1,800 mg/day) and measuring its effect on alcohol abstinence (60). The study shows that GBP significantly improved the rate of alcohol abstinence (*p* < 0.01), and no heavy drinking episodes were observed for this group. GBP also reduced relapse-related symptoms, such as insomnia, dysphoria, and craving, in addition to exhibiting a good safety profile.

Findings from numerous clinical trials suggest that GBP can be used as a monotherapy or as an adjunct therapy for alcohol withdrawal syndrome (AWS) treatment (56, 62), regardless of severity at presentation. GBP works effectively at high doses as a monotherapeutic for mild to moderate cases of AWS (56). However, it is also effective in severe cases when used as an adjunctive therapeutic agent. GBP has had much more success in the management of alcohol disorders than other conditions and is known to alleviate some symptoms of relapse, improving the rate of abstinence and delaying return to heavy drinking as documented by numerous clinical trials over the years (57–60).

#### ii Opioid Dependence

Pharmacotherapy for the treatment of chronic opioid addiction has focused on ameliorating withdrawal symptoms and reducing craving. The most commonly used approach to opioid detoxification includes the use of methadone, buprenorphine, and alpha_2_ adrenergic agonists such as clonidine or lofexidine (65). The exact mechanism is unknown, but one can hypothesize that GBP acts by inhibition of the neuronal Ca2+ channel. Opiate withdrawal syndrome is hypothesized to be due to the post-inhibitory excitatory syndrome. GBP enhances GABAergic activity or modulates the release of excitatory neurotransmitters because of its GABAergic properties (66). Our literature search found 6 RCTs, which studied the efficacy of GBP as an agent in opiate detoxification (66–71) (**Table 2**). GBP was found either alone or adjunctively to be significantly effective in achieving favorable changes in opiate detoxification symptoms and in reducing opioid consumption, e.g., Moghadam and Alavinia (69), Ziaaddini et al. (71), and Sanders et al. (70). An illustrative example is the Moghadam and Alavinia trial, where the efficacy of GBP was evaluated as an add-on agent for methadone detoxification (69). Sixty subjects were selected and were randomly assigned to two groups; group A was prescribed GBP and methadone (*N* = 34) and group B was given methadone and placebo (*N* = 26). The methadone dose was reduced in opium-addicted patients in group A. The results of the study showed that GBP is an effective add-on therapy when it is added to methadone. This drug leads to relief of withdrawal symptoms and lowers methadone consumption. GBP becomes a drug of interest when used as an adjunctive in treatment for opioid withdrawal based on its anti-anxiety, anticraving, and analgesic properties. The effectiveness of GBP in treating opioid dependence may lie in the dosage used within the clinical trials. Kheirabadi et al. found no significant benefits when treating 40 opioid-dependent subjects with 900 mg GBP (67). Contrary to those results, Salehi et al. conducted an open-label RCT to evaluate the efficacy of higher doses of GBP (1,600 mg) as an adjunctive medication in a standard methadone-assisted detoxification program (66). Compared to the previous trial with 900 mg/day GBP (67), the increased dose of 1,600 mg/day GBP was observed to be effective in reducing withdrawal symptoms in patients addicted to opiates.

#### iii Cocaine Dependence

Cocaine is a powerful stimulant of the sympathetic nervous system by inhibiting catecholamine reuptake, stimulating central sympathetic outflow, and increasing the sensitivity of adrenergic nerve endings to norepinephrine (NE). Cocaine causes irreversible structural changes on the brain, heart, lung, and other organs such as liver and kidney. Some of the adverse effects of cocaine include hepatotoxicity, coronary artery vasoconstriction, myocardial infarction, arrhythmias, myocarditis, ventricular hypertrophy, dilated cardiomyopathy, and heart failure. Cocaine demonstrated a high incidence of congenital cardiovascular and brain malformations in offspring born to mothers with a history of cocaine abuse. Because of the extreme effects of cocaine, very few of the medications currently available can combat cocaine craving and withdrawal symptoms. However, researchers are exploring different neurobiological modalities for cocaine dependence treatment. Because of the GABA-modulating effects of GBP, it is thought to be a likely candidate for ameliorating cocaine withdrawal symptoms and craving (72).

Six studies, comprising a total of 411 patients, have been performed in the format of an RCT or open-label trial (72–77). We reviewed these studies to evaluate the effect of GBP use on cocaine dependence. The majority of these trials found no significant benefit of GBP treatment compared with either the placebo or the other drugs tested in terms of the rate of abstinence achievement, retention in treatment, level of craving, or likelihood of future cocaine use (73–75, 77). Two open-label trials by Raby and Coomaraswamy (72) and Haney et al. (76) both showed some benefits of GBP in decreasing cocaine use and craving. However, these studies were very small (*N* = 9 and 8, respectively) (72, 76) in comparison with the much larger RCTs that concluded no benefit.

#### iv Amphetamine Dependence

Three trials have tested the efficacy of GBP in treating methyl amphetamine dependence (78–80) (**Table 2**). Two of these studies, Heinzerling et al. (78) and Ling et al. (79), concluded no significant benefit of GBP in the treatment of amphetamine dependence. Only the Urschel III et al. trial showed some positive benefit when GBP was used as an adjunctive (80). This trial tested a combination of flumazenil and GBP for treating methyl amphetamine dependence in 135 subjects over 30 days. Urine screens and self-reported drug usage indicated significantly decreased (*p* < 0.01) methyl amphetamine craving and use in the treatment group compared to the placebo group.

#### v Cannabis Dependence

Cannabis is one of the most widely used illicit drugs with a substantial proportion of the US population (81). A significant increase in the prevalence of marijuana use has been observed for the past decades across demographic subgroups. Cannabis use is associated with cognitive impairment, increased risk for psychotic disorders and other mental health problems, lower education attainment, and unemployment. Treatments for cannabis use disorder have likewise improved in recent years, focusing primarily on psychotherapy treatments, specifically motivational enhancement therapy, CBT, and contingency management. More recently, several pharmacotherapies have been explored through many clinical trials with some mixed results (82, 83). However, no pharmacologic treatment has emerged as clearly efficacious (84).

Mason et al. (85) RCT evaluated the efficacy of GBP in treating cannabis dependence. Fifty patients with cannabis dependence were randomly assigned to receive either the placebo or GBP (1,200 mg/day) over a period of 12 weeks. The researcher found that compared to the placebo, the GBP group had significantly reduced cannabis use as measured both by urine toxicology (*p* = 0.001) and by the Timeline Follow-Back Interview (*p* = 0.004). GBP also significantly reduced withdrawal symptoms as measured with the Marijuana Withdrawal Checklist (*p* < 0.001). GBP was associated with significantly greater (*p* = 0.029) improvement in overall performance when using tests of executive function. All subjects received weekly individual counseling as well (85). The study shows the clinical relevance of the effects of GBP on cannabis use and withdrawal, which is further supported by significantly greater improvement in executive function and psychological and physical problems with GBP compared to placebo. Although the study was able to show some evidence in support of GBP’s efficacy in treating cannabis dependence and withdrawal, the preliminary results from a proof-of-concept study should be taken cautiously for practical purposes.

## Discussion

There is growing evidence that GBP is effective in the treatment of various psychiatric and substance use disorders. However, GBP is currently approved for use only as an anticonvulsant and analgesic. With more than 18 million prescriptions of GBP per year, of which 83–95% are off-label, it is crucial that the use of GBP for these indications be supported by clinical data. In support of this, we carried out a systematic review of existing studies on the efficacy of GBP for the treatment of psychiatric disorders and comorbidities (86).

Our initial search of the PubMed and Ovid MEDLINE databases generated 2,604 results, which we successively filtered down to a final total of 54 studies using various inclusion/exclusion criteria. These studies were then classified based on the disorder being investigated and analyzed to extract the study findings. Overall, most of the studies, such as Pande et al. (36), Bonnet et al. (56), and Kheirabadi et al. (67) failed to show compelling evidence in support of GBP efficacy for the treatment of various psychiatric illnesses including some substance use disorders. While a small number of studies such as Anton et al. (53), Ménigaux et al. (11), and Moghadam and colleagues (69) indicated significant differences in the efficacy of GBP compared to placebo or control group. These studies reported some beneficial evidence in support of GBP's use in psychiatric disorders. The effectiveness of GBP may be accounted for by the different dosage regimens and augmentation with other medications. However, some discrepancies in published literature in regards to GBP's efficacy cannot be as easily validated, which may necessitate more rigorous studies on this subject.

Based on our review of the published literature and existing data, GBP appears to be effective in the treatment of various anxiety disorders of mild to moderate severity. Anxiety disorders such as generalized anxiety disorder, social phobia, and a fear of public speaking have been studies in patients with good clinical response to GBP (10, 12–14, 87). Also, GBP has been found effective in particular settings such as reduction of perioperative anxiety (7, 9, 11, 15, 16). The anxiolytic of GBP is established in both human and animal models studies (88). It has yet to be determined whether GBP is effective in treating severe and recurring anxiety. For bipolar disorder, GBP shows some level of effectiveness mainly as an adjunctive therapeutic treatment, while the evidence for monotherapy with GBP is inconclusive. Regarding substance disorders, the majority of GBP-related RCTs to date have evaluated the use of this drug in treating alcohol use disorders. GBP shows to be effective for acute alcohol withdrawal symptoms, cravings, sleeplessness, depression, and in maintaining abstinence. There are barriers to the effective use of FDA-approved agents in the management of alcohol use disorder including medical and psychiatric comorbidities, poor medication adherence, and problems with tolerability. As the body of research on alternatives to these agents continues to grow, it is crucial to understand the GBP’s strategic role in the management of alcohol dependence and withdrawal. GBP is not hepatically metabolized, making it potentially preferable to current FDA-approved agents, especially in this target population with a high prevalence of hepatic insufficiency. GBP can also be used for patients with renal function below 20 mg/dl, unlike acamprosate. Because of its fewer side effects, the added property of sleep improvement for patients with alcohol use disorders and the fact that it is generally well tolerated make GBP more promising and the favorable drug in this patient population. For the treatment of opioid abuse, GBP appears helpful in the treatment of opioid abuse as an adjunctive therapy and may also be effective as a monotherapy in treating cannabis dependence. However, this still lacks enough supportive evidence. No significant effect of GBP use has yet been conclusively observed in the treatment of OCD, PTSD, depression, or cocaine and amphetamine abuse; however, some of these disorders have not been investigated as in depth by larger randomized and controlled trials.

## Conclusion

Based on our review, GBP appears to be effective in the treatment of some forms of anxiety disorders and alcohol withdrawals or dependence. However, it is effective often as an adjunctive medication rather than as a monotherapy. More rigorous and larger clinical trials are required to resolve the contradictory existing data for the efficacy of GBP in the treatment of psychiatric and substance use disorders. It is worth noticing that numerous clinical studies that are discussed in this review are open-label trials, which are inherently less rigorously analyzed. Therefore, more extensive clinical studies are required to examine effectiveness of Gabapentin in both both psychiatric disorders and substance use disorders.

## Limitations

Further relevant studies may be present in databases other than the two used here. However, these two databases which we selected are the standard databases for archiving literature in this field. Our selected search terms may have also missed relevant studies. As discussed, there is a risk of bias in individual studies inherent in different dosage regimens and adopted outcome measurements; this further complicates the direct comparison of study outcomes. This study was a qualitative comparison rather than an in-depth meta-analysis.

## Disclosure

The findings of this review paper are provided for educational purposes. These findings suggest conclusions based only on a review of published data and should be interpreted cautiously in practical settings.

## Ethics Statement

The current review article identified published material and synthesized existing evidence that met pre-specified eligibility criteria to answer a specific question about the efficacy of Gabapentin. The study was entirely based on a review of previously published papers and didn’t require any consent from any human subjects. The findings and conclusion of this review paper based only on an analysis of published data and should be interpreted cautiously in practical settings in the best interest of patients.

## Author Contributions

All authors made substantial contributions to this study. SA, RB, PK, MA, and RA contributed to conception and design, analysis, and interpretation of data, and critical review of the manuscript. UF, HS, AK, AZ, IQ, and GB contributed to draft manuscript, the revision of the manuscript, advised on epidemiological methods, and was involved in the critical revision of the manuscript. SA, VK, RB, and MA contributed to the study conception. SA, RA, AZ, IQ, and GB contributed to the study design. SA, UF, HS, and RB contributed to the data analysis and interpretation. SA, MA, VK, RB, and UF wrote the paper draft. AZ, RA, IQ, HS, and VK contributed to the critical revision of the manuscript.

## Conflict of Interest Statement

The authors declare that the research was conducted in the absence of any commercial or financial relationships that could be construed as a potential conflict of interest.
